# A Comprehensive Propagation Prediction Model Comprising Microfacet Based Scattering and Probability Based Coverage Optimization Algorithm

**DOI:** 10.1155/2014/601729

**Published:** 2014-08-18

**Authors:** A. S. M. Zahid Kausar, Ahmed Wasif Reza, Lau Chun Wo, Harikrishnan Ramiah

**Affiliations:** Department of Electrical Engineering, Faculty of Engineering, University of Malaya, 50603 Kuala Lumpur, Malaysia

## Abstract

Although ray tracing based propagation prediction models are popular for indoor radio wave propagation characterization, most of them do not provide an integrated approach for achieving the goal of optimum coverage, which is a key part in designing wireless network. In this paper, an accelerated technique of three-dimensional ray tracing is presented, where rough surface scattering is included for making a more accurate ray tracing technique. Here, the rough surface scattering is represented by microfacets, for which it becomes possible to compute the scattering field in all possible directions. New optimization techniques, like dual quadrant skipping (DQS) and closest object finder (COF), are implemented for fast characterization of wireless communications and making the ray tracing technique more efficient. In conjunction with the ray tracing technique, probability based coverage optimization algorithm is accumulated with the ray tracing technique to make a compact solution for indoor propagation prediction. The proposed technique decreases the ray tracing time by omitting the unnecessary objects for ray tracing using the DQS technique and by decreasing the ray-object intersection time using the COF technique. On the other hand, the coverage optimization algorithm is based on probability theory, which finds out the minimum number of transmitters and their corresponding positions in order to achieve optimal indoor wireless coverage. Both of the space and time complexities of the proposed algorithm surpass the existing algorithms. For the verification of the proposed ray tracing technique and coverage algorithm, detailed simulation results for different scattering factors, different antenna types, and different operating frequencies are presented. Furthermore, the proposed technique is verified by the experimental results.

## 1. Introduction

Nowadays, indoor wireless communication becomes more and more popular in communication field. Because of increasing demand in this field, an effective propagation prediction technique and optimized coverage algorithm are required in order to support the demand by using the minimum number of transmitters (*T*_*x*_s) and at the same time achieving the maximum indoor wireless coverage. Though there are a number of existing research works based on ray tracing for propagation prediction [1–7], most of them have not mentioned anything about the coverage. Therefore, researchers are still in need of an efficient and integrated method, which can serve for propagation prediction and coverage optimization.

The main problem for the ray tracing based propagation prediction model is the ray-object intersection test. This test consumed the most time and resources in a ray tracing method [8, 9]. Intersection test is performed every time after a new ray is generated for finding whether there is a ray-object intersection or not. Hence, if all objects participate in this test, the ray tracing time consumed will be extremely high. To accelerate the ray tracing technique, various methods, such as angular sectoring [10], KD-tree, octree, quadtree [4, 5, 11], and a preprocessing method [8], are proposed. However, the existing models, such as shooting and bouncing ray (SBR) [4], bidirectional path tracing (BDPT) [12], brick tracing (BT) [13], ray frustums (RF) [14], prior distance measure (PDM) [8], and space division (SD) [15] techniques, require higher prediction time due to complex algorithms used and limitations of the used techniques. Moreover, the prediction accuracy is not so high. In SBR technique, double ray counting error occurs for the receivers (*R*_*x*_s) situated in two successive ray cone areas. The BDPT technique shows incorrect results for single floor multiple room environments and takes a lot of time to create the ray paths. The BT technique shows inaccuracy for corner bricks because of the truncation of the slab, which results in erroneous analytic reflection and transmission coefficient. In RF technique, a large computer memory is required for complex environments to store the huge amount of triangular frustums, which results in slow performance. In SD technique, all the IDs of the unique cells are stored in a single list and the full list has to search for finding a specific cell during simulation. This will consume a lot of time and increase the execution time. In PDM method, a preprocessing operation is needed for the environment. This type of preprocessing makes the overall process more complex. None of the above techniques are accompanied by the coverage optimization technique.

Considering all of the drawbacks of the existing techniques, this paper introduces a new method by including the rough surface scattering. This proposed ray tracing method is based on Adelson-Velskii and Landis (AVL) tree data structure, dual quadrant skipping (DQS) technique, and closest object finder (COF). The AVL tree has a lower data searching time and it is used for efficiently handling different information relative to the objects and environment. Both of the DQS and COF techniques are newly introduced and described here. Both of the techniques help to reduce the ray tracing time by eliminating the unnecessary objects and enhancing the ray-object intersection test. Furthermore, our proposed microfacet based scattering method is not dependent on specific environment and is able to figure out the scattering field in all likely directions. This makes the proposed ray tracing method more accurate.

Along with the ray tracing technique, a new coverage optimization algorithm is also introduced here where the probability is used to find out the most suitable *T*_*x*_ to be selected in order to achieve the optimum solution for indoor wireless coverage area. The probability of each *T*_*x*_ is directly affected by the *T*_*x*_ sampling pattern. The proposed algorithm introduces two types of probability that need to be taken into consideration, that is, intraprobability and interprobability. The concepts of probability will be explained in more detail in the next section. In order to support the probability concept, multilevel technique has been applied in the proposed algorithm where each *T*_*x*_ sampling pattern is viewed level-by-level instead of *T*_*x*_-by-*T*_*x*_. By applying multilevel technique in the proposed algorithm, it provided faster computation time; this is due to less *T*_*x*_ taking part in probability calculation for certain number of sampling points. For achieving better performance, genetic algorithm (GA) and depth first search (DFS) are used along with the probability theory for finding the optimum wireless coverage. GA is a widely used [16–19] algorithm for optimization of different electromagnetic problems. Here, it is used to optimize the number of *T*_*x*_s needed for covering the whole area. It will also optimize the position of the necessary *T*_*x*_s. In DFS, the node which is generated from the *E*-node is called a live node. *E*-node refers to the node where children are currently generated. The *E*-node is selected from various live nodes in the same level based on the probability. *T*_*x*_ having the highest probability will be selected as *E*-node and during each DFS process, every *T*_*x*_ can be selected as *E*-node at least once. To minimize the required computation time (time complexity) of the proposed coverage algorithm, changes have been made for the existing bounding and termination concepts that were proposed by the existing algorithm [19]. Basically, the bounding function uses for updating the latest probability of each new subbranch, hence improving the accuracy to determine the number of *T*_*x*_s required at corresponding positions. Besides, a termination criterion is used to avoid repeating select nodes, which had been selected as *E*-node before. The proposed algorithm generates less number of nodes in the search tree and further reduces the computation time by using this bounding function and termination criteria. Some analyses and comparisons have been made and the results prove that the proposed algorithm is more efficient than the existing algorithms [19, 20] in terms of space tree generated and also the time complexity.

## 2. Proposed Ray Tracing Technique

### 2.1. Technique for Achieving Balanced AVL Tree

For data storing and retrieving purpose, a dynamically height balanced binary search tree, named AVL tree, is used. An AVL tree has two basic properties: the height of the subtrees of every node differs by at most one and each subtree is an AVL tree. An AVL tree maintains a *O*(log⁡*n*) search time, while the addition and deletion operation also take *O*(log⁡*n*) time (where *n* is the number of objects). This timing is almost similar with another self-balancing tree, namely, red-black tree. However, the difference between them is the limiting height. For a tree of *n* objects, the maximum height of an AVL tree is strictly less than [21]

(1) logφ(5(n+2))−2=log2(5(n+2))logφ−2=logφ(2)log2(5(n+2))−2≈1.44log2(n+2)−0.328,

where *φ* is the golden ratio.

At the same time, the maximum height of a red-black tree is [22]

(2) $$\begin{array}{c} h=2\text{lo}{\text{g}}_{2}\left(n+1\right). \end{array}$$

Therefore, we can say that the AVL trees are more rigidly balanced than red-black trees. For this reason, the AVL tree data structure is chosen.

The data insertion technique in an AVL tree is identical to a binary search tree, where it is done by expanding a peripheral node. For maintaining balance, information of the balance factor will have to be stored in every node. This balance factor will maintain the balance efficiently after each insertion or deletion operation. The balance factor, bf, can be represented as

(3) bf=height  of  left  subtree (hLsubtree) −height  of  right  subtree (hRsubtree).

This bf indicates if the two subtrees are in the same height or not. The bf of a height balanced binary tree can be one of the values −1,0, +1. An AVL node is called left heavy when bf = −1, equal heavy when bf = 0, and right heavy when bf = +1. After insertion of each new node, the balance factors have to be updated. If the balance factor becomes less than −1 or greater than +1, the tree becomes unbalanced and rotation process will be undertaken for making balanced AVL tree.

**Figure 1:**
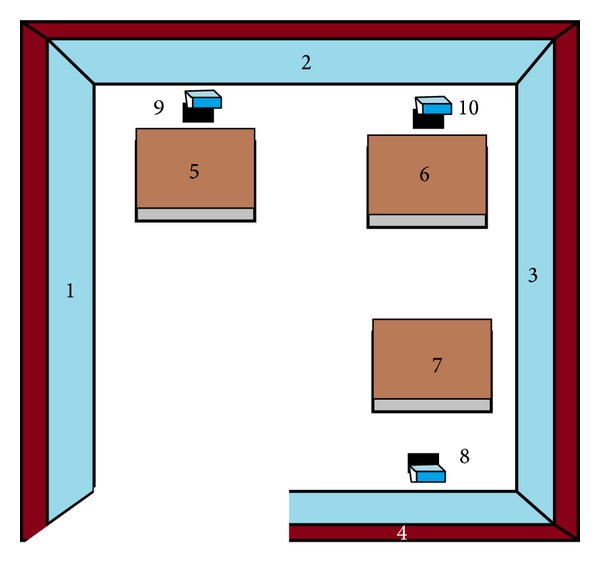
Sample environment for showing the AVL tree creation.

**Figure 2:**
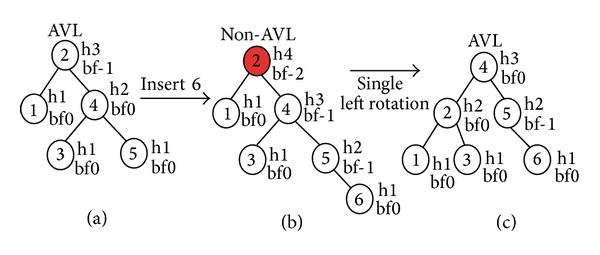
Balancing of AVL tree by a single rotation: (a) insertion of new node, (b) non-AVL tree, and (c) balanced AVL tree after single rotation.

**Figure 3:**
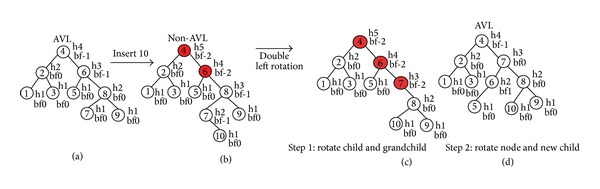
Balancing of AVL tree by double rotation: (a) insertion of new node, (b) non-AVL tree, (c) 1st rotation, and (d) 2nd rotation and balanced AVL tree.

Here, Figure 1 represents a sample environment of 10 objects. The creation of AVL tree from this sample environment is presented here in Figures 2 and 3.  Figure 2 shows a single rotation for making balance after insertion of 6th object and Figure 3 shows balancing with double rotation after insertion of the 10th object in the sample environment of Figure 1. The further insertion of objects can be done by following this technique.

### 2.2. Microfacet Based Scattering Model

**Figure 4:**
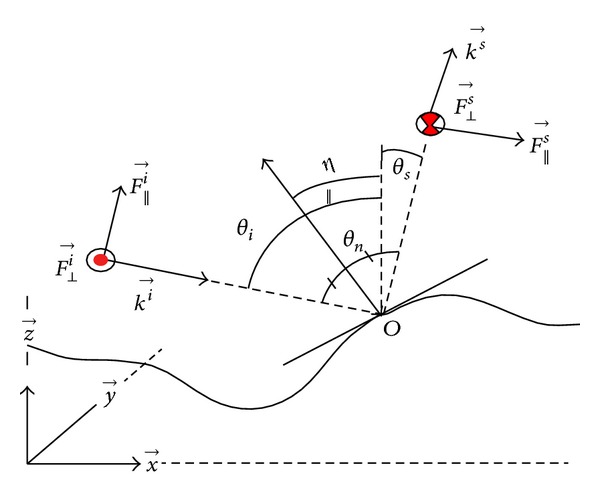
Notations used in the KA based scattering model.

In this section, the computational model for computing the scattering field of rough surface has been presented. This model is based on Kirchhoff approximation (KA) [18, 23], which is a well-known method. Hence, the rough surface is decomposed into small planes that are nearly tangent to the roughness and these are called microfacets.  Figure 4 represents the profile of a rough surface, a random tangent plane on it, and the notations, which are going to be used for incident and scattered fields.

An incident plane wave irradiates the decomposed rough profile. Thus, the same amount of incident plane wave is received by each microfacet and the surface reflects it towards its own specular direction. As well as the smooth surface, this specific specular direction can be defined by incident angle *θ*_*i*_ and alignment of the indigenous normal *η* of a microfacet.

Therefore, the scattering field can be computed as

(4) $$\begin{array}{c} {\vec{F}}_{//,⊥}^{s}=R_{//,⊥}\left(\theta_{i},\theta_{s}\right){\vec{F}}_{//,⊥}^{i}e^{-j\varphi }e^{-j(\phi_{i}+\phi_{s})}, \end{array}$$

where ${\vec{F}}_{//,⊥}^{s}$ is the scattered field and ${\vec{F}}_{//,⊥}^{i}$ is the incident field in both polarizations, *R*_//,⊥_(*θ*_*i*_, *θ*_*s*_) is the Fresnel reflection coefficient between incident *θ*_*i*_ and scattered *θ*_*s*_ directions, and *e* ^−*j*(*ϕ*_*i*_+*ϕ*_*s*_)^ is the shifting of phase caused by the free space propagation distance after *ϕ*_*s*_ and before *ϕ*_*i*_. The term *e* ^−*jφ*^ represents the shifting of phase because of the height *h* of the microfacet regarding the global mean value, which is usually set at *h* = 0 [12]. It can be written as

(5) $$\begin{array}{c} \varphi =\frac{4\pi }{\lambda }h\cos\left(\theta_{i}\right). \end{array}$$

Now, a rational sum of all the aid of the scattered fields summarized in a minute solid angle *dθ* around a definite direction *θ*_*s*_ will have to be formulated. In reality, the orientation of many microfacets can be the same but, depending on the global mean value, their heights are not automatically same. If *n* numbers of microfacets among *N* numbers are well directed, the rational sum is equivalent to a vector sum for each component $\left(\vec{x},\vec{y},\vec{z}\right)$ of the scattered fields [12]:

(6) $$\begin{array}{c} {\vec{F}}_{//,⊥}^{s}=\overset{n}{\underset{i=1}{\sum }}{\vec{F}}_{//,⊥,i}^{s}, \end{array}$$

where *F*_//,⊥,*i*_^*s*^ is the scattering field for both of the polarizations of the *i*th microfacet.

As a result of the plane wave propagation condition, the simplified scattering field for the *i*th microfacet can be written as

(7) $$\begin{array}{c} {\vec{F}}_{//,⊥\cdot i}^{s}=R_{//,⊥,i}\left(\theta_{i},\theta_{s}\right)e^{-j\varphi_{i}}{\vec{F}}_{//,⊥,i}^{i}\underset{=C^{te}}{\underset{︸}{e^{-j(\phi_{i}+\phi_{s})}}}\;. \end{array}$$

Now, the whole scattering field around a particular direction will be expressed for the parallel element. The similar interpretation can be used for the perpendicular polarization. So, the scattering field of the *i*th microfacet in parallel polarization can be expressed as

(8) $$\begin{array}{c} {\vec{F}}_{//,i}^{s}\left(\theta_{s}\right)=\{\begin{array}{cc} R_{//}\left(\theta_{i},\theta_{s}\right)\frac{1}{N}\cos\left(\theta_{s}\right)e^{-j\varphi_{i}}C^{te} & \vec{x}\\ 0 & \vec{y}\\ R_{//}\left(\theta_{i},\theta_{s}\right)\frac{1}{N}\sin\left(\theta_{s}\right)e^{-j\varphi_{i}}C^{te} & \vec{z}. \end{array} \end{array}$$

Then, the vector sum in *dθ* around *dθ*_*s*_ direction for *n* contributions becomes

(9) $$\begin{array}{c} {\vec{F}}_{//,i}^{s}\left(\theta_{s}\right)=\{\begin{array}{cc} C^{te}R_{//}\left(\theta_{i},\theta_{s}\right)\frac{1}{N}\cos\left(\theta_{s}\right)\overset{n}{\underset{i=1}{\sum }}\left\{e^{-j\varphi_{i}}\right\} & \vec{x}\\ 0 & \vec{y}\\ C^{te}R_{//}\left(\theta_{i},\theta_{s}\right)\frac{1}{N}\sin\left(\theta_{s}\right)\overset{n}{\underset{i=1}{\sum }}\left\{e^{-j\varphi_{i}}\right\} & \vec{z}. \end{array} \end{array}$$

If we set up the ratio *n*/*n* in (9), the scattering field will become

(10) $$\begin{array}{c} \left|{\vec{F}}_{//}^{s}\left(\theta_{s}\right)\right|=R_{//}\left(\theta_{i},\theta_{s}\right)C^{te}\frac{n}{N}\frac{1}{n}\overset{n}{\underset{i=1}{\sum }}e^{-j\varphi_{i}}, \end{array}$$

where the ratio *n*/*N* signifies the probability of having *n* number of microfacets from *N* possible numbers in *θ*_*s*_ direction and (1/*n*)∑_*i*=1_^*n*^ *e* ^−*jφ*_*i*_^ represents the mean attenuation due to the shifting of phase. Therefore, the scattering field of (10) can be written with replacement of *n*/*N* by the probability density function *p*(*θ*_*s*_) as

(11) $$\begin{array}{c} \left|{\vec{F}}_{//}^{s}\left(\theta_{s}\right)\right|=R_{//}\left(\theta_{i},\theta_{s}\right)C^{te}p\left(\theta_{s}\right)\frac{1}{n}\overset{n}{\underset{i=1}{\sum }}e^{-j\varphi_{i}}. \end{array}$$

The scattering coefficient *σ* ^//,⊥^(*θ*_*s*_) can be deduced from (11) for both polarizations, which provides the ratio between the scattered power in a solid angle *dθ* around *θ*_*s*_ and the incident power:

(12) $$\begin{array}{c} \sigma^{//,⊥}\left(\theta_{s}\right)=\left|{\vec{F}}_{//,⊥}^{s}\left(\theta_{s}\right)\right|{\left|{\vec{F}}_{//,⊥}^{s}(\theta_{s})\right|}^{*}. \end{array}$$

### 2.3. Proposed Optimization Techniques for Ray Tracing

After creating the data structure tree, it is necessary to find the objects, which are taking part in intersection test. We have sorted the necessary objects in two different techniques. First, we have used the proposed DQS technique to find a group of objects according to the ray direction. Then, the COF will find the nearest object from that particular group of objects and that nearest object will take part in intersection test. These two acceleration techniques will reduce the intersection test time by finding the exact object.

**Figure 5:**
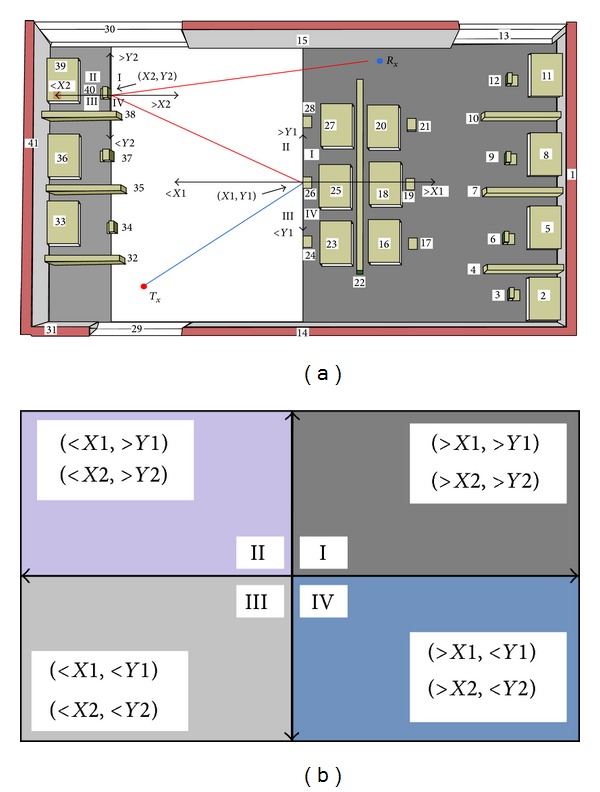
(a) Object skipping using DQS technique in a sample environment and (b) object allocation due to DQS.

The DQS technique will reduce the prediction time by reducing the number of considered objects due to each intersection test. From Figure 5, we can easily describe the DQS technique. Suppose a ray is generated from the *T*_*x*_ and intersects with an object at the position (*X*_1_, *Y*_1_). According to the position of the objects in the environment with reference to the point (*X*_1_, *Y*_1_), the environment will be divided into four quadrants: I, II, III, and IV. The distribution of the objects into different quadrants is illustrated in Figure 5.

Now assume the object is a nontransparent object and no diffraction is occurring. Hence, the ray will show normal reflection or scattered reflection. Therefore, there is no possibility for the reflected ray to go behind the object. That means that, for the next intersection test, we have no need to consider the objects of quadrants I and IV. Thus, all of the objects in the regions I and IV will be skipped for the next intersection test. This saves almost half of the prediction time for the next intersection test. Now assume, after reflection of the ray at (*X*_1_, *Y*_1_), the ray intersects with another object at the position (*X*_2_, *Y*_2_). Again, the whole environment will be divided into four quadrants based on (*X*_2_, *Y*_2_). After reflecting on (*X*_2_, *Y*_2_), the ray will go to the front of that object. Again, the shaded portion of the back side of that object will be skipped, which means quadrants II and III will be skipped. If there is refraction (for transparent object) or diffraction instead of reflection, the opposite portions of the quadrant (quadrants II and III) will be skipped and thus will reduce the prediction time.

By applying the DQS technique, a group of possible intersecting objects can be found and, among them, some objects (e.g., 39th, 40th, and 41st) are parallel. All of them will not intersect with the ray, but only the nearest object will take part in the intersection test. To find the nearest object, we have introduced the COF. The COF is composed of two different phases: first, the creation of an effective artificial surface (EAS) inside each of the possible intersecting objects found by DQS and second finding the distances between the ray source and different EAS by using the “Pythagoras Theorem.”

**Figure 6:**
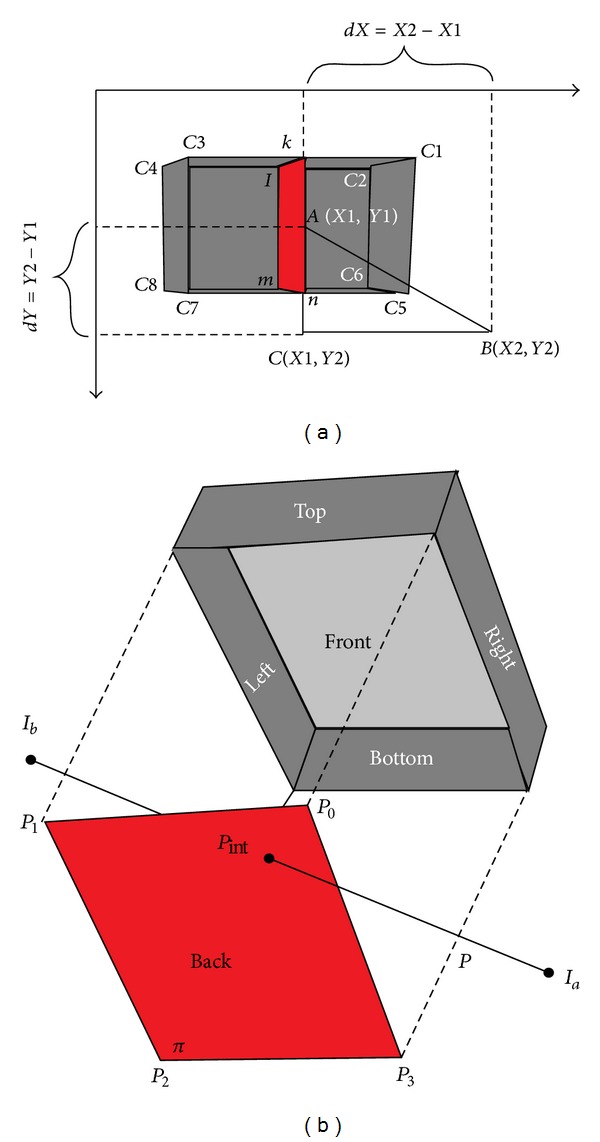
(a) Distance calculation between ray source and EAS. (b) Intersection point calculation between ray and a surface.

The EAS as shown in Figure 6(a) is defined as the effective surface within an object, which is used for calculating ray-object intersection. In this concept, a new invisible or imaginary surface is created inside the 3D object at a point where the ray contacted with the object. This surface will be a 2D surface and it is created at the middle of the object. It is introduced with the aim of simplifying the algorithm and, hence, reducing the computational complexity. Using the six vertex points of a cuboid, the software developed in this work will calculate the coordinates of the EAS.

Assuming *k*, *l*, *m*, and *n* are the four vertices of the EAS and *C*_1_,  *C*_2_, *C*_3_, *C*_4_, *C*_5_, *C*_6_, *C*_7_, and *C*_8_ are the eight vertices of the cube or cuboids, then the coordinate of *k* of the ICS can be found as

(13) $$\begin{array}{c} X_{k}=\frac{X_{C_{1}}-X_{C_{3}}}{2},\\ Y_{k}=Y_{C_{1}}=Y_{C_{3}}, \end{array}$$

and *l*, *m*, and *n* coordinates are determined in the same manner as the *k* coordinate.

Now, if the coordinate of *k* is (*x*_1_, *y*_1_) and coordinate of *n* is (*x*_2_, *y*_2_), then the coordinate (*X*_1_, *Y*_1_) of the middle point of *kn* face will be as

(14) $$\begin{array}{c} X_{1}=\frac{\left(x_{1}+x_{2}\right)}{2},\\ Y_{1}=\frac{\left(y_{1}+y_{2}\right)}{2}. \end{array}$$

Now, suppose the middle of the EAS of the 39th, 40th, and 41st objects is *A*, *D*, and *E*, respectively, and (*X*_1_, *Y*_1_) is the coordinate of *A*. These points will be used for calculating the distance between the objects and ray source point (*X*_2_, *Y*_2_). This will be done by using the “Pythagoras Theorem.”

By extending the points *A* and *B*, a right angled triangle *ABC* will be formed (Figure 6(a)). The coordinates of the point *C* will be (*X*_1_, *Y*_2_). Now, by applying the “Pythagoras Theorem” in the right angled triangle, we found

(15) $$\begin{array}{c} AB^{2}=AC^{2}+BC^{2}={\left(Y_{2}-Y_{1}\right)}^{2}+{\left(X_{2}-X_{1}\right)}^{2}. \end{array}$$

If the distance between *A* and *B* is *D*_1_, then, from (15), we found

(16) $$\begin{array}{c} D_{1}=\sqrt{dX^{2}+dY^{2}}, \end{array}$$

where *dX* is the difference between the *X*-coordinates of *A* and *B* and *dY* is the difference between the *Y*-coordinates of *A* and *B*.

By this way, distance between *B* and other middle points will be calculated, which can be *D*_2_ and *D*_3_, respectively, in the present case. Then, after comparing the distances, the nearest object is chosen and, in the above case, 40th object is found as the nearest. As the nearest object, this one will now be used for an intersection test to find the intersection point. After finding the nearest object, the intersection test is done for getting the exact ray-object intersection point. Based on this intersection point, the next ray shooting decision will be taken from this point. The occurrence of reflection or refraction is also dependent on this point.

As we have used 3D cuboids for representing the objects, there is a possibility of intersection between the ray and the six faces of the cuboids. Depending on the position of the object with respect to the ray shooting point, the exact face is chosen for finding the intersection point. As an example, if an object is in the first quadrant then the ray will possibly hit the left or back surface of that object and in that case, only these surfaces will be used for finding the intersection point, as demonstrated in Figure 6(b). Similarly, right or back surface for the second quadrant, right or front surface for the third quadrant, and left or front surface for the fourth quadrant, respectively, will be used. In case of the 34th object of Figure 5, right or back surface will have to be tested for finding the intersection point and it is shown in Figure 6(b). According to vector notation, the plane *π* is expressed as the set of points *P* for which

(17) $$\begin{array}{c} \left(P-P_{0}\right)\cdot n=0, \end{array}$$

where *n* is a normal vector to the plane and *P*_0_ is a point on the plane.

According to vector algebra, the line of the ray can be expressed as

(18) $$\begin{array}{c} P=dI+I_{0},\;d=R, \end{array}$$

where *I* is a vector in the direction of the line, *I*_0_ is a point on the line, and *d* is a scalar in the real number domain.

Now, by substituting (18) into (17), we get

(19) $$\begin{array}{c} \left(dI+I_{0}-P_{0}\right)\cdot n=0. \end{array}$$

By distributing *n*, (19) becomes

(20) $$\begin{array}{c} dI\cdot n+\left(I_{0}-P_{0}\right)\cdot n=0. \end{array}$$

Now, by solving (20), we get the value of *d*:

(21) $$\begin{array}{c} d=\frac{\left(P_{0}-I_{0}\right)\cdot n}{I\cdot n}. \end{array}$$

No intersection will exist if the line is parallel to the plane and starts outside the plane. In this case, (21) denominator will be zero and the numerator will be nonzero. The line will intersect everywhere with the plane if the line is parallel to the plane and starts inside the plane. In this case, both the numerator and denominator of (21) will be zero. In all other cases, the line intersects the plane once and *d* represents the intersection as the distance along the line from *I*_0_; that is, *dI* + *I*_0_. Now, this intersection point (*P*_int⁡_(*X*, *Y*, *Z*)) will act as the source for that particular ray and reflection, refraction, diffraction, or scattering will occur at that point according to the object property and the ray will proceed to a particular direction. Based on that direction, again, DQS will apply at the intersection point and the next object will be found for a ray-object intersection by applying the COF technique. This process will continue and, at the end, the ray will be counted either as a valid signal received by *R*_*x*_ or an invalid signal.

### 2.4. Formulation of Time Complexity and Received Power

As we have mentioned earlier, for *M* number of objects, the search operation of an AVL tree can be implemented in *O*(log⁡*n*) time. Based on (1), the time complexity of the proposed technique can be described as below.

Let *M* be the number of objects, let *N* be the number of surfaces of each 3D object, and let *S* be the intersection testing time for the proposed method. If *R* numbers of intersections are required to predict each significant ray, then the total intersection testing time can be calculated by the following equation:

(22) $$\begin{array}{c} S_{\text{proposed}}=R\times N\times O\left(\text{lo}{\text{g}}_{2}M\right). \end{array}$$

Moreover, according to DQS, the proposed method can omit a significant amount of objects during each intersection test. Let *M*_DQS_ be the average number of omitted objects due to DQS. Now, (22) becomes

(23) $$\begin{array}{c} S_{\text{proposed}}=R\times N\times O\left(\text{lo}{\text{g}}_{2}\left(M-M_{\text{DQS}}\right)\right). \end{array}$$

Furthermore, the COF technique also skips objects during intersection test. Suppose *M*_COF_ is the number of objects skipped by COF technique. Thus, the equation for intersection time will be

(24) $$\begin{array}{c} S_{\text{proposed}}=R\times N\times O\left(\text{lo}{\text{g}}_{2}\left(M-M_{\text{DQS}}-M_{\text{COF}}\right)\right). \end{array}$$

For similar cases, the intersection testing time by using the existing techniques [12, 24, 25] can be found as

(25) $$\begin{array}{c} S_{\text{existing}}=R\times N\times O\left(M\right). \end{array}$$

Accordingly, the time complexity of SBR technique is [16]

(26) $$\begin{array}{c} S_{\text{SBR}}=R\times N\times O\left(\text{lo}{\text{g}}_{2}\left(M-M_{\text{MT}}\right)\right),\; \end{array}$$

where *M*_MT_ are the skipped objects due to mailbox technique.

Time complexity for BT and BDPT techniques can be found as [16]

(27) $$\begin{array}{c} S_{\left(\text{BT}\&\text{BDPT}\right)}=R\times N\times O\left(M\right). \end{array}$$

The time complexity of RF technique is [16]

(28) $$\begin{array}{c} S_{\text{RF}}=R\times N\times O\left(\text{lo}{\text{g}}_{2}\left(M-2^{H}\right)\right), \end{array}$$

where 2^*H*^ is the order of quadtree.

Time complexity for PDM and SD techniques is [16]

(29) $$\begin{array}{c} S_{\text{PDM}}=R\times N\times O\left(M-M_{\text{PDM}}-M_{\text{BSM}}\right), \end{array}$$

where *M*_PDM_ and *M*_BSM_ are the objects skipped due to prior distance measures and bounding spheres method, respectively.

And

(30) $$\begin{array}{c} S_{\text{SD}}=R\times N\times O\left(M-M_{\text{SD}}\right), \end{array}$$

where *M*_SD_ are the objects skipped by space division.

The received power at an observation point is calculated with Friis transmission formula. For three-dimensional modeling, three-dimensional directivity data of transmitting and receiving antennas are required, which can be interpolated from the measurement data along the *E*- and *H*-planes [26].

If an observation point is included in a ray frustum, a ray path is formed, which traces back to the vertex of the frustum. If the ray hits the reflecting triangle or the diffracting edge that generates the frustum, the ray continues to trace back to the apex of the parent ray frustum. The process is recursively carried out until the ray hits the transmitting antenna. If the ray is intercepted by obstacles in the course of the ray tracing, the received power at the point is assumed to be zero. When the back-traced ray hits the source, the directivity *n* of the transmitting antenna along the direction of the ray is utilized and the radiated electric field is obtained by [14].

Consider

(31) $$\begin{array}{c} E_{\text{inc}}=\sqrt{\frac{\eta P_{T}}{4\pi }G_{T}\left(\theta_{T},\phi_{T}\right)}.\frac{h_{T,\nu }{\hat{\theta }}_{T}+h_{T,h}{\hat{\phi }}_{T}}{R}e^{-jkR}, \end{array}$$

where *P*_*T*_ is the transmitted power and *η* is the wave impedance of the free space. The gain along the ray direction (*θ*_*T*_, *ϕ*_*T*_) is *G*_*T*_(*θ*_*T*_, *ϕ*_*T*_). Unit vectors ${\hat{\theta }}_{T}$ and ${\hat{\phi }}_{T}$ represent the vectors along the elevation and azimuth directions, respectively. The values *h*_*T*,*v*_ and *h*_*T*,*h*_ represent the polarization components.

The incident electric field generated from the transmitting antenna undergoes reflection, transmission, and diffraction. The received electric field of a ray that arrives at the receiving antenna after many combinations of reflections, diffractions, and transmissions can be found as

(32) $$\begin{array}{c} E_{\text{received}}=\left[\prod A\left(S_{i-1},S_{i}\right)\left({\overset{-}{R}}_{i}\;\text{or}\;{\overset{-}{T}}_{i}\;\text{or}\;{\overset{-}{D}}_{i}\right)e^{-jkS_{i}}\right]\cdot E_{\text{inc}}, \end{array}$$

where *A*(*S*_*i*−1_, *S*_*i*_) is the divergence factor that accounts for the magnitude changes after *i*th scattering. The dyadic quantities ${\overset{-}{R}}_{i}$, ${\overset{-}{T}}_{i}$, and ${\overset{-}{D}}_{i}$ are reflection, transmission, and diffraction.

The received voltage is the sum of multipath signals that have propagated along actual ray paths. The voltage can be obtained as

(33) $$\begin{array}{c} V_{R}=\lambda \sqrt{\frac{Z_{0}}{4\pi }\sum G_{R}\left(\theta_{R},\phi_{R}\right).\left[\left(h_{R,\nu }{\hat{\theta }}_{R}+h_{R,\nu }{\hat{\phi }}_{R}\right)\cdot E_{\text{received}}\right]}, \end{array}$$

where *Z*_0_ is the characteristic impedance of a receiver and *G*_*R*_(*θ*_*R*_, *ϕ*_*R*_) is the gain along the ray direction (*θ*_*R*_, *ϕ*_*R*_). Unit vectors ${\hat{\theta }}_{R}$ and ${\hat{\phi }}_{R}$ represent vectors along the elevation and azimuth directions seen from the receiving antenna coordinate, respectively. The values *h*_*R*,*v*_ and *h*_*R*,*h*_ represent the polarization components. The received power can be obtained as

(34) $$\begin{array}{c} P_{R}=\frac{{\left|V_{R}\right|}^{2}}{Z_{0}}. \end{array}$$

## 3. Proposed Coverage Algorithm

The basic idea of the proposed algorithm is to obtain the minimum number of *T*_*x*_s required to achieve the optimum wireless coverage. To achieve this goal, the concept of *T*_*x*_s' probability to be selected for each sampling point has been introduced and incorporated with GA and DFS to optimize the indoor wireless coverage. Here, a sampling point is a point where it collects the signals coming from the surrounding *T*_*x*_s. Ray tracing is used to generate different coverage patterns.

In this study, the DFS progresses by expanding the first level of sampling point nodes of the tree that appear and finding out the node with a higher probability and searching from level to level until all the sampling point is covered; then, searching process will be stopped. The key word of the proposed algorithm is the probability. For ease of understanding, the used notations are given here.

- *t*_*i*_ is the *i*th *T*_*x*_. Assume each of the *T*_*x*_s only covers one of the sampling points *S*. Therefore, the number of *T*_*x*_s required to cover all the sampling* points* should be equal to or less than the total number of sampling points. Hence, 1 ≤ *i* ≤ *n*, if the number of sampling points in the indoor area is *n*.
- *C*_*i*_ is the coverage pattern of *t*_*i*_.
- *L*_*i*_ is the sampling point level for level *i*th.
- *P*_*ij*_ is the probability of *t*_*i*_ to be selected for *L*_*j*_.
- *T*_*P*_*i*__ is the total probability to be selected for *t*_*i*_.
- *R*_*i*_ is the *t*_*i*_ to be removed.

If *C*′and *C*′′ are the two coverage patterns, then the resultant pattern C* will be as

(35) $$\begin{array}{c} C^{*}=\left\{C^{\prime }\cup C^{\prime \prime }\;|\;\overset{n}{\underset{i=1}{\sum }}e_{i}\geq 0\right\}. \end{array}$$

As an example, if 8 sampling points are numbered from 1 to 8 and one *T*_*x*_ covers the sampling points 1, 2, 5, and 7, respectively, and another *T*_*x*_ covers the sampling points 2, 3, 7, and 8, respectively, then the coverage pattern for that *T*_*x*_ will be as in Table 1. Here, the resultant pattern C* is created by merging both *C*_1_ and *C*_2_ based on the concept of logical inclusive “OR” operation, as shown in Table 1, where the result is “1,” if the first bit is “1” OR the second bit is “1” or both bits are “1.” Otherwise, the result is “0.”

The probability of *T*_*x*_ is based on a few principles.

(i) If the number of *e*_*i*_ covered by the *T*_*x*_ becomes higher, then the term for summation will increase. Therefore, the probability of the *T*_*x*_ to be selected will become higher. This is named as intraprobability. Intraprobability for *T*_*x*_ is
  (36) $$\begin{array}{c} t_{i}=\overset{n}{\underset{i=1}{\sum }}\text{multiplication}\;\text{term}\;\text{of}\;P_{ij}. \end{array}$$
(ii) If the numbers of *T*_*x*_s which cover the same sampling point become smaller, then the probability of the *T*_*x*_ to be selected for that particular sampling point level will become higher. This is named as interprobability. Interprobability for each sampling point level is
  (37) $$\begin{array}{c} P_{ij}=\frac{1}{\left(\sum_{i=1}^{n}t_{ie_{j}=1}\right)}. \end{array}$$
(iii) The probability for each *T*_*x*_ can be equal to 1, if and only if the *T*_*x*_ consists of a sampling point which is not covered by other *T*_*x*_s; otherwise, the probability of the *T*_*x*_ should be always smaller than 1.
(iv) If the probability is equal to 1, the integer value shows the number of sampling points that are only covered by this *T*_*x*_ and not other *T*_*x*_s.
(v) Only the maximum probability of each *T*_*x*_ *P*_*i*_ will be taken.

For example, if there are 6 sampling points from 1 to 6, the coverage pattern of these six *T*_*x*_s will be as shown in Table 2.

The first level sampling point, *L*_1_, is only covered by *t*_1_, *t*_2_, and *t*_3_; therefore, the probability for *L*_1_, *P*_11_, will be 1/3. By using the same principle, the probability of *L*_2_, *L*_3_, *L*_4_, *L*_5_, and *L*_6_ is obtained, as shown in Table 2. By using the above data, we found the probability for the *t*_1_ as 0.74074 (*T*_*P*_1__ = *P*_13_ + *P*_13_∗*P*_11_ + *P*_13_∗*P*_11_∗*P*_12_ + *P*_13_∗*P*_11_∗*P*_12_∗*P*_14_) and the probability for *t*_4_ as 1.3333 (*T*_*P*_4__ = *P*_46_ + *P*_46_∗*P*_45_).

Some of the bounding functions as well as termination criteria are applied while going deeper into the search space. For *n* number of sampling points in the indoor environment, the proposed bounding functions are as follows.

- The probability of each existing and nonselected *T*_*x*_ for each sampling point level is recalculated, where the calculation starts from the current sampling point level up to *n*th level of sampling point. Therefore, the probability of each node will be reduced from sampling point level to level.
- The process needs to be run for *n* + 1 times, by removing one of the *T*_*x*_s each time. The step is used to make sure the chosen *T*_*x*_s are the minimum number of *T*_*x*_s required to achieve the optimum wireless coverage. Besides, this step is also able to obtain another alternative for choosing *T*_*x*_ without degrading the coverage.

For making the proposed algorithm complete, the following termination criteria have been used. The termination happens if one of the following conditions becomes true.

- The existing *T*_*x*_ in current sampling point level is one of the chosen *T*_*x*_s in previous sampling point level. Termination happens to the current sampling point level and proceeds to the next level.
- Algorithm termination happens when no live node exists in the solution space to be explored.

**Figure 7:**
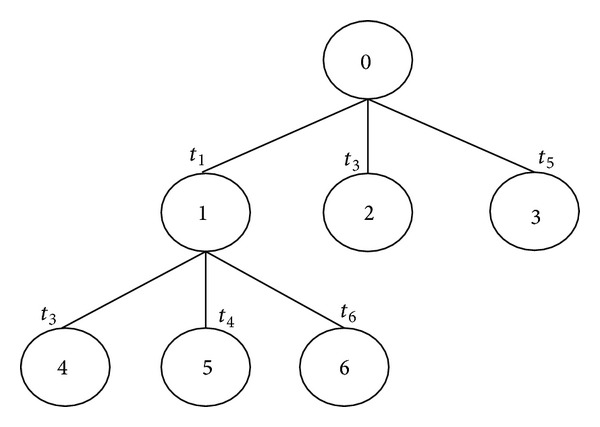
State space search tree generated by the proposed algorithm.

To illustrate the proposed method, suppose there are 6 sampling points in the indoor propagation area and the coverage pattern is shown in Table 2.  Figure 7 generates the state space search tree based on sampling point level to achieve the first optimal solution.

Initially, there is only one live node, node 0. This represents the case in which no *T*_*x*_ has been placed in the propagation area. This node becomes the *E*-node at the initial state. It is expanded based on sampling point level *T*_*x*_; therefore, for sampling point level, *L*_1_, its child nodes 1, 2, and 3 are generated. These nodes represent the solution space, where only one *T*_*x*_ is considered at a time. By calculating the probability of *t*_1_, *t*_3_, and *t*_5_ as shown in Table 2, the *t*_1_ has a higher probability; therefore, the next *E*-node will be node 1 and the algorithm switches to the next sampling point level. Since, in sampling point levels 2, 3, and 4, *t*_1_ exists again, according to the termination criteria, all these levels will be terminated and the *E*-node will remain as 1.

For 5th sampling point, only 3rd, 4th, and 5th *T*_*x*_s exist in this sampling point level, by applying the first bounding function, where the probability of these *T*_*x*_s will be recalculated, starting from the current sampling point level. Since sampling point level *L*_6_ is only covered by 6th *T*_*x*_, therefore, according to the third probability principle, the probability for 4th *T*_*x*_ is equal to 1. Since, in last sampling point level, *t*_4_ exists again, the termination criteria will terminate this level and the *E*-node will remain as 5. Then, the resultant coverage pattern is as follows:

(38) C∗=C1∪C4=1 ∣ 1 ∣ 1 ∣ 1 ∣ 1 ∣ 1 .

**Figure 8:**
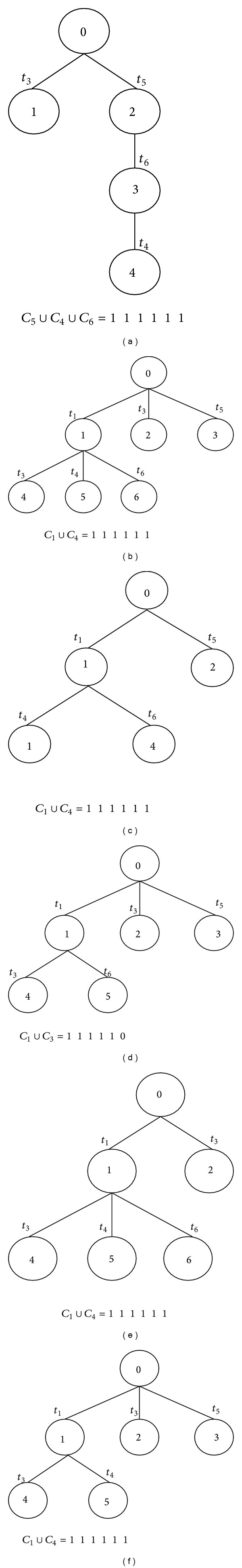
Different stages of getting optimum solution. *T*_*x*_ removed (a) *R*_1_, (b) *R*_2_, (c) *R*_3_, (d) *R*_4_, (e) *R*_5_, and (f) *R*_6_.

The above pattern shows the primary optimum solution will be formed by the 1st *T*_*x*_ and 4th *T*_*x*_. By applying the second bounding function, the proposed algorithm will be rerun again by removing one of the *T*_*x*_s each time; therefore, the algorithm needs to run for 7 times in order to get the correct optimum solution by comparing the latest coverage pattern with the primary optimum solution obtained and also the number of *T*_*x*_s required.  Figure 7 is the first step and Figure 8 represents the rest of the steps.

### 3.1. Complexity Analysis

In this section, comparison between the proposed and existing algorithms [19, 20] is presented in terms of both space and time complexities. Here, the received signal at each sampling point due to one or more *T*_*x*_s is calculated by using the ray tracing algorithm. Initially, for the worst case, each *T*_*x*_ covers one sampling point; therefore, *n* numbers of *T*_*x*_s are needed for *n* number of sampling points. The ray tracing algorithm runs for every sampling point from the location of the *T*_*x*_ to generate the coverage pattern for each *T*_*x*_. At the beginning, the signal is transmitted from a particular *T*_*x*_ situated at a certain location (e.g., *A*) to each of the sampling points. If the signal is able to be detected by another *T*_*x*_ situated at another particular sampling point (e.g., *B*), then the signal generated by the *T*_*x*_ of location *B* should also be able to reach the *T*_*x*_ located at *A*. Therefore, the number of ray tracing algorithms to be run decreases for each *T*_*x*_. For example, if there are *n* numbers of sampling points, then 1st *T*_*x*_ is required to run ray-tracer *n* − 1 times and 2nd *T*_*x*_ will run *n* − 2 times, and so on. Therefore, the total amount of ray tracing algorithms to be run for *n* numbers of *T*_*x*_s is

(39) $$\begin{array}{c} \left(n-1\right)+\left(n-2\right)+\left(n-3\right)+\cdots +3+2+1=\overset{n}{\underset{i=1}{\sum }}n-i. \end{array}$$

**Figure 9:**
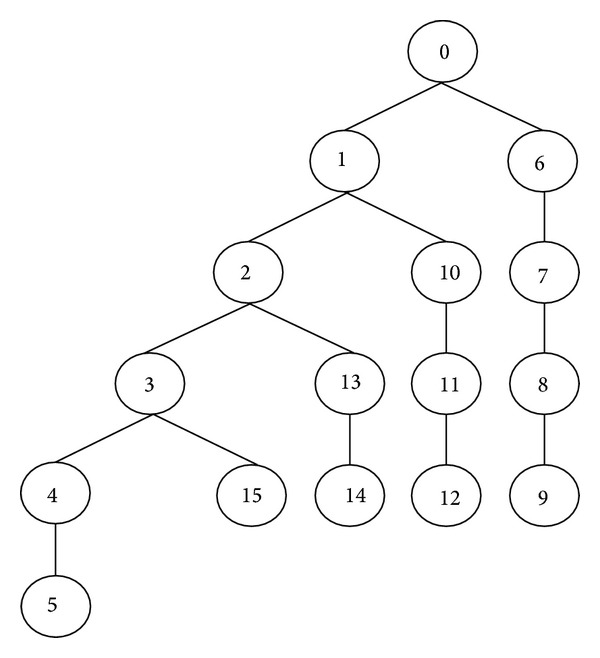
Tree organization for the proposed coverage algorithm.

The proposed algorithm requires all *T*_*x*_ sampling pattern information in order to perform the probability calculation. Hence, before proceeding to the proposed algorithm, the ray tracing algorithm needs to run completely for generating sampling pattern for all *T*_*x*_s. Based on this algorithm, for a typical indoor environment having 5 sampling points, the worst case search tree can be generated, as shown in Figure 9.

Node 0 refers to only the root node of the tree, so it does not refer to any *T*_*x*_. Therefore, node 0 of the tree can be referred to as dummy node. By referring to the tree organization of Figure 9, the total space tree required is

(40) $$\begin{array}{c} \text{Total}\;\text{space}\;\text{tree}=6+5+4+3+2+1+\text{dummy}\;\text{node}. \end{array}$$

It can be further simplified by writing the sum of the natural numbers up to *n* in two ways as

(41) $$\begin{array}{c} S_{n}=1+2+3+\cdots +\left(n-2\right)+\left(n-1\right)+n,\\ S_{n}=n+\left(n-1\right)+\left(n-2\right)+\cdots +3+2+1. \end{array}$$

Adding these two series,

(42) $$\begin{array}{c} 2S_{n}=\left(n+1\right)+\left(n+1\right)+\cdots +\left(n+1\right). \end{array}$$

There are *n* of these (*n* + 1)'s, so

(43) $$\begin{array}{c} 2S_{n}=n\left(n+1\right),\\ S_{n}=\frac{n\left(n+1\right)}{2}. \end{array}$$

Hence, the sum of the natural numbers from 1 to *n* is therefore half the product of the first term plus the last one multiplied by the number of terms. Thus, in general, the total number of nodes generated by the tree can be calculated as the following formula:

(44) $$\begin{array}{c} \frac{n\left(n+1\right)}{2}+\text{dummy}\;\text{node}=\frac{n\left(n+1\right)}{2}+1, \end{array}$$

where *n* is the number of sampling points in the selected indoor environment. As all the generated nodes stay in memory, therefore, the space complexity is also the same as the time complexity. The space complexity refers to the number of nodes generated until the deepest level, and the time complexity depends on the number of nodes generated until the required solution has been found [19].

The proposed algorithm will be compared with the existing algorithms [19, 20]. According to existing algorithm [20], the time complexity can be expressed as

(45) 1+∑r=1n ∑i=11(r−i)+∑r=2n ∑i=12(r−i)+∑r=3n ∑i=13(r−i)   +⋯+∑r=n−1n ∑i=1n−1(r−i)+∑r=nn ∑i=1n(r−i)  =1+∑r=1nC0+∑r=2nC1+∑r=3nC2+⋯+∑r=n−1nCn−1+∑r=nnCn  =2n,

where *r* is the number of rows and *n* is the number of sampling points in the indoor environment. Equation (45) represents the time complexity of the existing algorithm [20] for the worst case.

The time complexity of the existing algorithm [19] can be expressed as follows by modifying existing algorithm [20]:

(46) $$\begin{array}{c} 2^{n-m}-k, \end{array}$$

where *n* is the number of sampling points in the selected indoor environment, *m* is the number of coverage patterns that has been rejected because of duplication, and *k* is the number of nodes that has been unexplored. In the worst case, the value of both *m* and *k* will be 0.

From Table 3, it is clear that both time and space complexities of the proposed algorithm are better than the existing algorithms [19, 20]. From the table, it is also apparent that the time and space complexities of the existing algorithms are increasing exponentially while the space and time complexity of the proposed algorithm increase linearly. Therefore, we can say that the proposed algorithm will take significantly lower execution time than the existing algorithms.

## 4. Results and Discussion

For fair comparison, the same environments are used and all experimental settings have been kept equivalent. All data in this section have been taken with the same simulation setup. All of the comparable techniques (in a format which gives the best possible outcome) have been implemented in the same simulation software. All simulations are carried out on a PC consisting of Intel(R) Core(TM) 2 Duo CPU E8400 @ 3.00 GHz, 3.23 GB of RAM, operated by Microsoft Windows XP operating system. Thus, there is no chance of discriminations between the techniques. Though this proposed technique is applicable for any frequencies, in most of the cases, 2.4 GHz operating frequency and half wave dipole antenna with 2.15 dBi gain have been used. The objects of the simulation software were made by using cuboids. The properties of different materials are used to make the simulation environment the same as realistic environments. The material properties are shown in Table 4.

**Figure 10:**
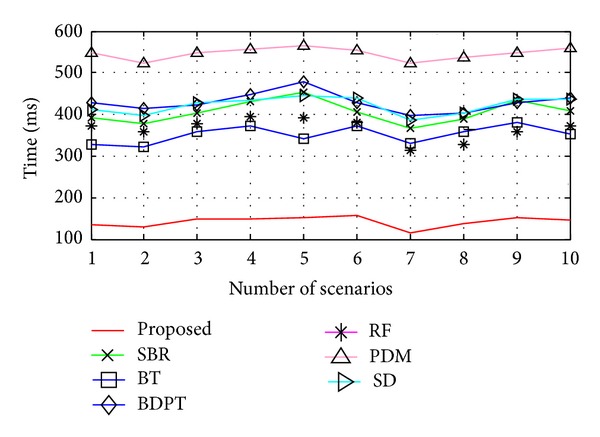
Comparison in terms of time.

To evaluate the performance of the proposed technique, a comparison is made with the existing methods. The comparison is made between the proposed technique and the SBR, BT, BDPT, RF, PDM, and SD techniques. The drawbacks of the existing techniques have been described in Section 1. For proper comparison, five different environments are chosen (one of them is shown in Figure 5). The environments are different by means of a number of objects. Some are mostly complex and some are moderate. Measurements are done in 10 different sampling points for each environment, by changing the *T*_*x*_ and *R*_*x*_ positions. The results obtained from 10 different sampling points of Figure 5 are represented graphically in Figure 10.  Table 5 represents the overall results for all five environments.

According to Figure 10, the proposed algorithm shows lower time consumption for the environment shown in Figure 5. Results for five such kinds of different environments are represented in Table 5. From the results, we observe that the proposed algorithm shows 69.93% lower time consumption than SBR algorithm, 63.67% lower than BT, 82.44% lower than BDPT, 66.03% lower than RF technique, 83.36% lower than PDM method, and 82.12% lower than SD technique. This is because the DQS helps to decrease the time by skipping objects during simulation, the COF technique minimizes the ray-object intersection time, and the AVL tree data structure minimizes the data searching time.

**Figure 11:**
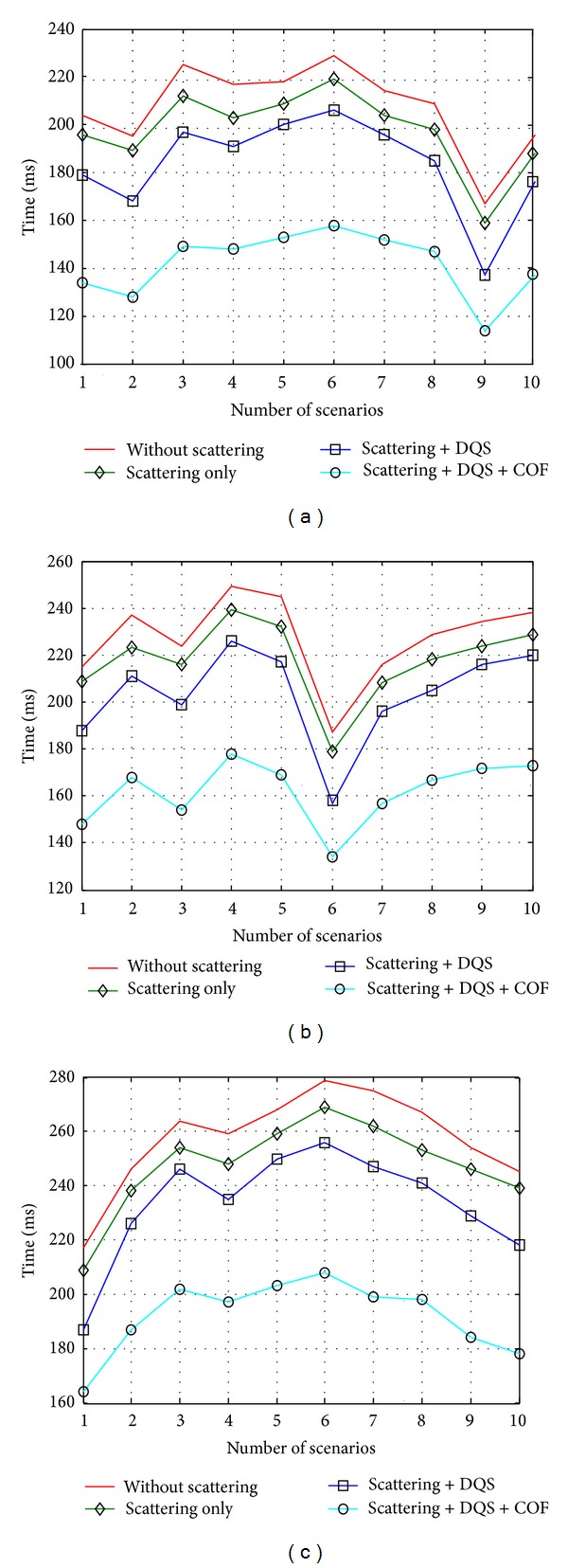
Effects of scattering and the proposed optimization techniques on time for (a) SF = 4, (b) SF = 8, and (c) SF = 20.

For the justification of the inclusion of rough surface scattering and different optimization techniques, this part presents the effects of rough surface scattering and the proposed optimization techniques on prediction time. These effects are presented graphically in Figure 11 for different scattering factors (SF). SF is the key feature which has impact on scattering simulation. SF has a key impact on scattering angle. When the SF increases, the scattering angle also increases. Thus, the chance of ray-object interaction increases and it increases the prediction time. Here, a comparative study is presented to show the improvement of the proposed technique step-by-step. For SF = 4 (Figure 11(a)), after including the scattering in the ray tracing technique, the time decreases by 4.65% in average compared to the algorithm that has not considered scattering in ray tracing. Furthermore, 11.68% time reduction is achieved for the inclusion of DQS optimization technique and 31.55% reduction for the inclusion of both DQS and COF techniques. For SF = 8 (Figure 11(b)) and SF = 20 (Figure 11(c)), the time also decreases step-by-step after including the scattering and different optimization techniques. These results demonstrate strong effects of scattering and optimization techniques on prediction time.

**Figure 12:**
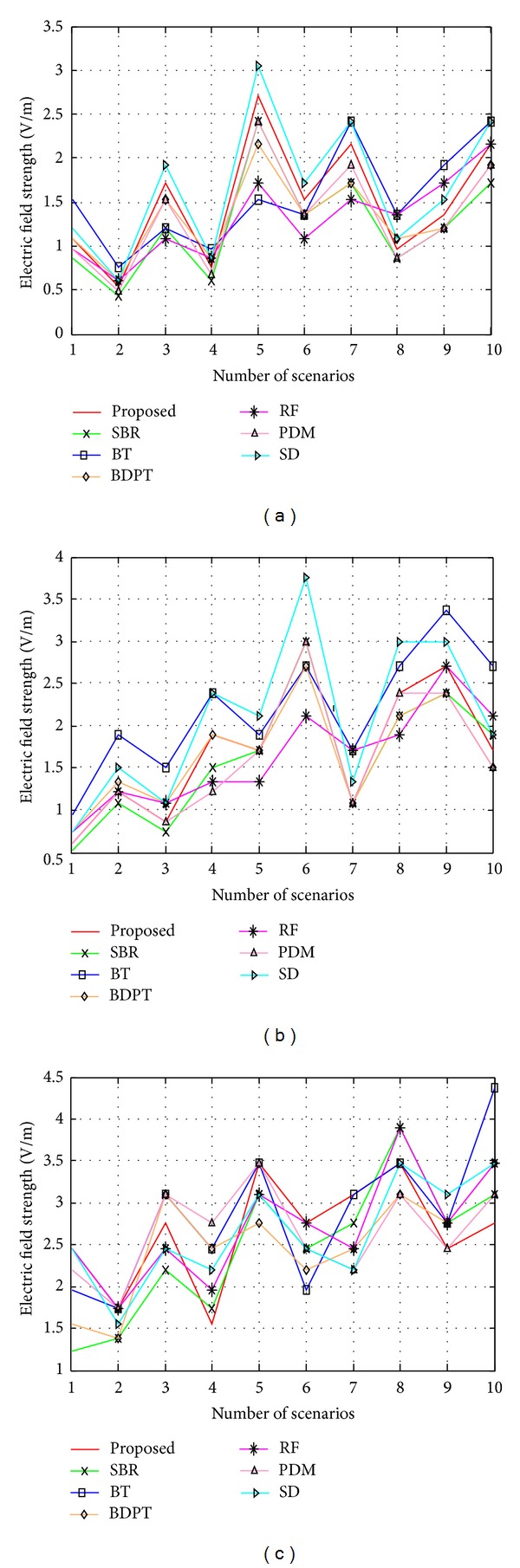
Electric field strength with (a) half wave dipole antenna, (b) bow-tie antenna, and (c) hemispherical antenna.

As we know, there are a number of types of antennas, which can be used as *T*_*x*_ and *R*_*x*_. For different types of antennas, the received power and electric field strength will be different due to different antenna characteristics (i.e., antenna gain).  Figure 12 shows three different antennas and the electric field strength in the receiver side. Here, the antennas used are half wave dipole antenna with 2.15 dBi gain, bow-tie antenna with 2.3 dBi gain, and hemispherical antenna with 7 dBi gain. In all cases, 2.4 GHz operating frequency is used for data collection. In the figures, almost all of the techniques show nearly similar field strength at the receiver end. There are small differences between the techniques and this difference is a result of multipath propagation, which is represented by different approaches in different algorithms. As a result, variation occurs in the electric field strength at the receiver end. Another cause of the variation is different optimization techniques used in the algorithms. Due to these optimization techniques, the amount of the received signal varies and, thus, the electric field strength also varies. This nearly similar result of different techniques verifies the validity of the proposed technique.

**Figure 13:**
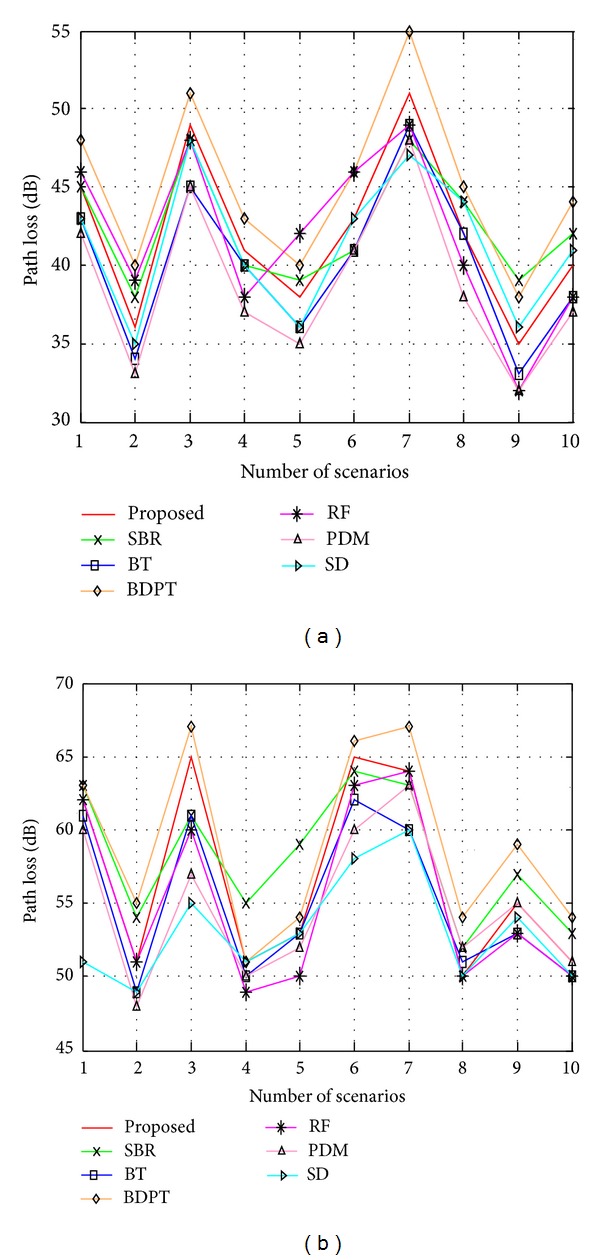
Comparison between the proposed and existing algorithms based on the average path loss for (a) 900 MHz and (b) 2.44 GHz.

In the above comparisons, verification is done by the data taken using different antennas with the same operating frequency. In this part, the same thing is done by changing the operating frequency for the same antenna to observe the effect of different frequencies. As it is known that the operating frequency has an impact on the path loss calculation [27], Figure 13 represents the path loss data for half wave dipole antenna in two different frequencies. 900 MHz frequency is used for Figure 13(a) and 2.4 GHZ is used for Figure 13(b). For 900 MHz, the path loss varies from 38.8 dB to 45 dB and for 2.4 GHz, it varies from 53.1 dB to 59 dB for different techniques. It indicates that, due to increasing the operating frequency, the path loss also increases.

**Figure 14:**
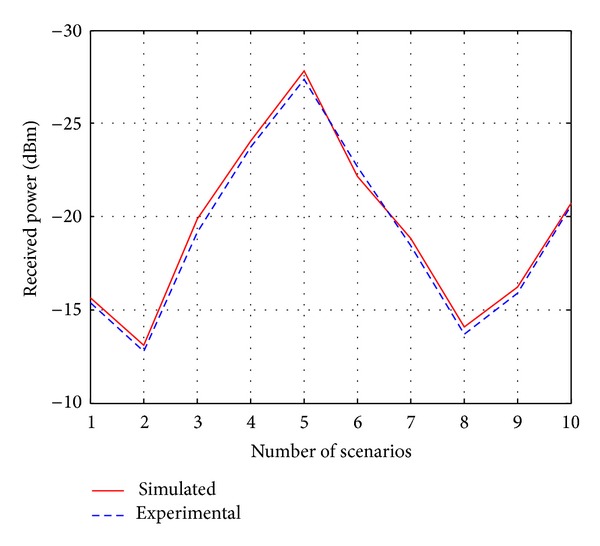
Received power in different scenarios.

In previous discussions, the validity and the superiority of the proposed ray tracing technique are done with the simulation results. However, in this part, the comparison between the simulated and the experimental data is also shown in order to evaluate the validity of the proposed technique. In real cases, the transmitted power is constant and the received power is variable for different *R*_*x*_s depending on the *T*_*x*_-*R*_*x*_ separation, the amount of obstacles, and so forth. Based on the received power, the signal strength of *R*_*x*_ changes, which makes the received signal a “good” signal or a “bad” signal. For this reason, the received power has significance in ray prediction and we have chosen this parameter for the comparison.  Figure 14 shows the comparison between the simulated and experimental received power for different scenarios. During experiment and simulation, the transmitted power is kept constant. From the figure, it is observed that the simulation results are almost matching with the experimental results, which validate the proposed algorithm. The average difference between the simulation and experimental data is very small and it is 1.65%. These differences are the effects of different environmental parameters, object properties, and the existence of other electromagnetic signals in the practical environment.

**Figure 15:**
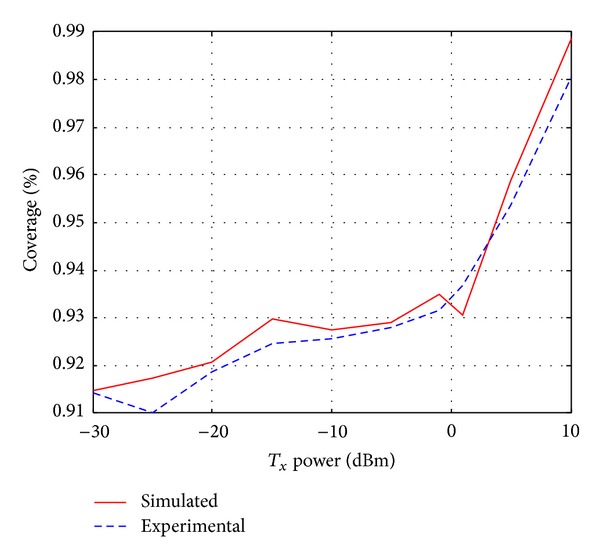
Comparison between simulated and experimental coverage.

Until now, it is apparently found that the proposed ray tracing technique is valid and more efficient than the existing ray tracing techniques. Here, validation of the proposed coverage algorithm is deliberated in comparison with the simulated and experimental results for different transmitted power. The coverage for a specific environment is changeable with the changes in transmitted power. If the transmitted power is constant, the coverage will depend on the obstacles. If the number of obstacles or position of obstacles changes, it will make changes in the coverage. Therefore, in case of constant transmitted power, the coverage will remain almost the same for the same environment. For this reason, we have verified our algorithm with different transmitted power for the same environment and achieved approximately 99% and 98% maximum coverage in terms of simulation and experiment, respectively, for 10 dBm transmitted power (refer to Figure 15). Also, from Figure 15, we observe the matching results between the simulation and experimental results of the coverage algorithm. The average difference between the simulation and experimental data is 0.3%. This difference is the result of different environmental effects and trivial mismatching between the simulation and experimental setup. These matching results confirm the accuracy of the proposed coverage algorithm.

## 5. Conclusion

A new propagation prediction technique for indoor environment is proposed in this paper where AVL tree is used for data storing and retrieving process, and DQS and COF techniques are used for accelerating the overall ray tracing process. This paper also proposed a new algorithm for wireless indoor coverage, which is based on the probability theory and optimized by GA and DFS. Analysis between the proposed method and the existing methods proved that the proposed method has lower time and space complexities. The obtained results reveal that the proposed technique improved the performance in terms of lower computational time of about 83.36%. In case of coverage algorithm, the space and time complexities of the proposed algorithm are greatly improved because of strong bounding functions and termination criteria as well as the concept of multilevel technique for finding the suitable *T*_*x*_s from the coverage pattern based on the probability. The similarities between the simulated and experimental results (a very high matching accuracy of more than 98% with respect to received power and coverage) confirm the validity of the proposed technique.

## Figures and Tables

**Table 1:** Coverage pattern for 8 sampling points.

		*e*_1_	*e*_2_	*e*_3_	*e*_4_	*e*_5_	*e*_6_	*e*_7_	*e*_8_
		↓	↓	↓	↓	↓	↓	↓	↓
*C*_1_	=	1	1	0	0	1	0	1	0
*C*_2_	=	0	1	1	0	0	0	1	1
C*	=	1	1	1	0	1	0	1	1

**Table 2:** Example of sampling pattern for 6 *T*_*x*_s.

	*e*_1_	*e*_2_	*e*_3_	*e*_4_	*e*_5_	*e*_6_
*t*_1_=	1	1	1	1	0	0
*t*_2_=	0	1	0	1	0	0
*t*_3_=	1	0	0	0	1	0
*t*_4_=	0	0	0	0	1	1
*t*_5_=	1	1	0	0	0	0
*t*_6_=	0	0	1	1	1	0
*P*_*ij*_	1/3	1/3	1/2	1/3	1/3	1

**Table 3:** Complexities of the existing and the proposed methods.

Complexity	Existing [20]	Existing [19]	Proposed
Space	2^*n*^	2^*n*−*m*^ − *k*	*n*(*n* + 1)/2 + 1
Time	2^*n*^	2^*n*−*m*^ − *k*	*n*(*n* + 1)/2 + 1

**Table 4:** Properties of different materials.

Material	Dielectric constant, *εr*	Refractive index, *η*
Brick	5.2	
Glass	3	1.52
Wood	3	
Plastic board (PVC)	4	1.46

**Table 5:** Combined results for all five environments.

		Proposed	SBR	BT	BDPT	RF	PDM	SD
Time (ms)	1	112	407	352	528	365	646	521
	2	76	247	204	487	221	492	476
	3	69	231	192	473	211	479	461
	4	85	269	216	502	242	511	498
	5	105	328	265	545	275	551	534
